# Monoclonal Antibodies Targeting CGRP to Treat Vestibular Migraine: A Rapid Systematic Review and Meta-Analysis

**DOI:** 10.1007/s12070-024-04578-y

**Published:** 2024-03-11

**Authors:** Andrea Frosolini, Andrea Lovato

**Affiliations:** 1 Department of Medical Biotechnologies, Maxillofacial Surgery Unit, University of Siena, Viale Mario Bracci n13, Siena, 53100 Italy; 2 Department of Surgical Specialties, Otorhinolaryngology Unit, Vicenza Civil Hospital, Vicenza, Italy; 3 Department of Surgical Specialties, Otorhinolaryngology Unit, San Gaetano Clinic, Thiene, Vicenza Italy

**Keywords:** Vestibular migraine, Monoclonal antibodies, CGRP, Systematic review, Meta-analysis, Migraine disability, Treatment efficacy, Vestibular symptoms, Vestibular disorders

## Abstract

Vestibular migraine (VM), a subtype of migraine characterized by vestibular symptoms, poses a significant diagnostic and therapeutic challenge. This study aimed to evaluate the effectiveness of monoclonal antibodies targeting Calcitonin Gene Related Peptide (CGRP) in the treatment of VM. Therefore, we conducted a rapid systematic review and meta-analysis following PRISMA and Cochrane guidelines. A search of databases (PubMed, Scopus, Cochrane and Google Scholar) was performed in October 2023. Inclusion criteria required original research articles focusing on patients diagnosed with VM and utilizing CGRP-targeting monoclonal antibodies. We performed qualitative assessments of study design, patient characteristics, and outcomes and, for studies with comparable outcome measures, a meta-analysis was conducted. Our search yielded four relevant studies, including cohort studies and a case report, totaling 99 patients. Proper vestibular instrumental tests were employed in half of the studies. Overall, the included studies reported significant improvements in VM symptoms. Our quantitative analysis, focused on migraine symptoms, demonstrated a substantial reduction in Monthly Days with Migraine at 6 months following treatment. No severe adverse drug reactions were reported. In conclusion, this rapid systematic review and meta-analysis provide preliminary evidence for the efficacy of CGRP-targeting monoclonal antibodies in treating Vestibular Migraine. However, the absence of randomized controlled trials and variations in study designs and diagnostic criteria introduce some limitations. Further research is needed, including controlled trials, to establish a more robust evidence base. Nonetheless, this treatment approach offers hope for the effective management of VM, potentially enhancing the well-being of affected individuals and reducing their associated disability.

## Key Points

1. **Novel Therapeutic Strategy**: Monoclonal antibodies targeting CGRP offer a promising new treatment avenue for Vestibular Migraine.
2. **Significant Symptom Improvement**: Patients administered with these antibodies exhibited notable reductions in the frequency and severity of symptoms.
3. **Migraine-related Disability**: There’s a marked decrease in migraine-related disability among patients treated with monoclonal antibodies against CGRP.
4. **Research Gaps Identified**: Despite the promising results, there’s a lack of randomized controlled trials and a considerable heterogeneity among existing studies.
5. **Call for Further Research**: High-quality, randomized controlled trials are urgently needed to solidify the role of these antibodies in clinical management of Vestibular Migraine.

## Introducition

Vestibular migraine (VM), a subtype of migraine characterized by vestibular symptoms, is one of the most frequent outpatients diagnoses in vestibology [1]. Diagnostic criteria for VM were established in 2012, requiring at least five episodes of moderate to severe vestibular symptoms lasting 5 min to 72 h, with a history of migraine, accompanied by at least one migraine headache feature in at least 50% of episodes, and exclusion of other headache or vestibular disorders [1]. VM manifests with various forms of vertigo and auditory symptoms, affecting approximately 1–2.7% of adults and often co-occurring with migraines. Differential diagnosis of VM can be challenging due to shared clinical features (i.e. Ménière’s disease), necessitating a comprehensive diagnostic approach [Lovato et al. 2021]. Several medications have been considered for VM management, randomized control trials are scarce and clear recommendations are lacking [2–5]. Recent studies suggested that CGRP antagonism may alleviate neuronal hyperactivity and maladaptivity in VM [6, 7]. The Calcitonin Gene Related Peptide (CGRP), a 37-amino acid neuropeptide, interacts with the calcitonin receptor-like receptor and receptor activity-modifying protein to exert vasodilatory effects and play a role in cardiovascular regulation, wound healing, and pain pathways. In the context of migraine and VM, CGRP is implicated in pathophysiological mechanisms, including ion channel changes and neurotransmitter release [8]. Additionally, CGRP has been targeted successfully with monoclonal antibodies (mAbs) for migraine prevention, significantly improving patients’ quality of life and reducing disability [9]. There is preliminar evidence regarding the efficacy of anti-CGRP mAbs in VM prevention [10]. Therefore, considering the need for research on new treatments for VM, we conducted a rapid systematic review and meta-analysis with the objective to evaluate the efficacy of monoclonal antibodies targeting CGRP in the treatment of VM.

## Materials and Methods

### Electronic Database Search

The present rapid systematic review was conducted according to the PRISMA statement and following the Cochrane rapid review methods guidance [11]. A search strategy to identify relevant studies was employed on the following databases: PubMed, Scopus, Cochrane and Google Scholar. To capture studies specifically related to the use of CGRP-targeting monoclonal antibodies in the context of VM, we used a Boolean search strategy, combining the terms “CGRP” and “Vestibular Migraine.” All the aforementioned databases were searched on day 18 October 2023 without time restrictions. The related search option on the database website and the reference list of all included manuscripts were screened for eligibility of additional potential relevant studies.

### Inclusion and Exclusion Criteria

The following inclusion criteria were applied: (i) original research articles; (ii) patients diagnosed with VM; (iii) use of monoclonal antibodies targeting CGRP for the treatment of VM. The following exclusion criteria were applied: (i) non original research (e.g. review); (ii) non english lenguage study. The studies that did not meet these criteria were excluded.

### Data Extraction and Manuscript Assessment

To identify potentially relevant articles, two independent reviewers conducted an initial screening of titles and abstracts (A.F. and A.L). Subsequently, full-text articles of potentially eligible studies were retrieved and subjected to detailed review. Data extraction was conducted independently by both reviewers, and any discrepancies encountered during this process were resolved through discussion and consensus. A Qualitaty assessment of included studies was done according to the Joanna Briggs Institute (JBI) clinical appraisal tool for cohort studies [12].

### Qualitative and Quantitative Analysis

Our analysis comprised both qualitative and quantitative assessments of the included studies. We assessed the included studies focusing on study design, patient characteristics, intervention details, and outcome measures. We extracted findings related to the efficacy and safety of monoclonal antibodies targeting CGRP in the context of VM. For studies with comparable outcome measures, we conducted a meta-analysis using appropriate statistical methods, such as random-effects models. This quantitative analysis allowed us to generate pooled effect sizes, confidence intervals, and forest plots, providing a comprehensive assessment of the overall treatment effect of CGRP-targeting monoclonal antibodies in the management of Vestibular Migraine. The statistical analysis was performed with the MetaHun Software Version 2.0 [13].

**Table 1:** Evaluation of included manuscript according to the Joanna Briggs Institute (JBI) clinical appraisal tool for cohort studies

	Russo			Hoskin			Lovato		
	Yes	No	NA	Yes	No	NA	Yes	No	NA
Were the two groups similar and recruited from the same population?			x			x			x
Were the exposures measured similarly to assign people to both exposed and unexposed groups?			x			x			x
Was the exposure measured in a valid and reliable way?	x				x		x		
Were confounding factors identified?		x			x			x	
Were strategies to deal with confounding factors stated?		x			x			x	
Were the groups/participants free of the outcome at the start of the study (or at the moment of exposure)?			x			x			x
Were the outcomes measured in a valid and reliable way?		x			x		x		
Was the follow up time reported and sufficient to be long enough for outcomes to occur?	x				x		x		
Was follow up complete, and if not, were the reasons to loss to follow up described and explored?	x				x		x		
Were strategies to address incomplete follow up utilized?		x			x			x	
Was appropriate statistical analysis used?	x			x			x		
Total:	4/8			1/8			5/8		

## Results

### Retrieved Studies

**Fig. 1:**
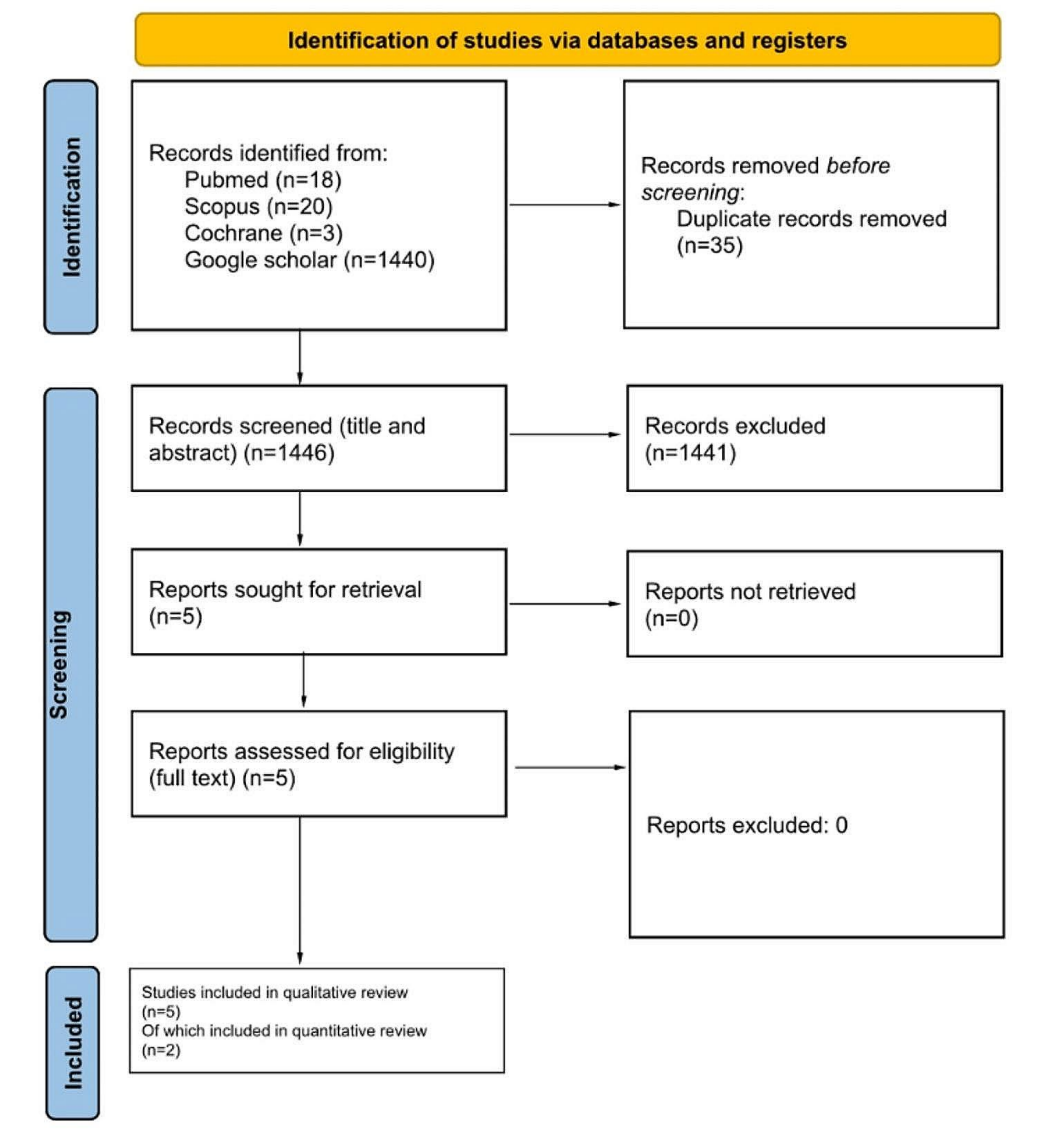
Study selection from identification to inclusion according to the PRISMA steatment

After database searching, 1446 articles were retrieved. Following application of inclusion and exclusion criteria, four original studies [14–17] were finally included in the present systematic review as shown in the PRISMA diagram (see Fig. 1). Three manuscripts were cohort studies with both prospective [16] and retrospective design [14–16], one was a case report [17]. Overall quality ranged from fair [14, 16] to low [15], as shown in Table 1. Moreover, a RCT protocol with enrollment ending in July 2023 registered on ClinicalTrials.gov (official website of the U.S. Department of Health and Human Services, National Institutes of Health, National Library of Medicine, and National Center for Biotechnology Information) was found by searching the Cochrane database. The double blinded RCT aims to investigate the efficacy of Galcanezumab in treating VM, evaluating changes in VM-Patient Assessment Tool and Handicap Inventory scores as a primary outcome measure in 50 patients. Galcanezumab, will be administered through subcutaneous injections over four months, and its impact will be compared to a placebo [18].

### Qualitative Analysis

Table 1 summarizes the main characteristics and findings of the four included studies, overall reporting 99 patients. The diagnosis of VM was made according to the International Headache Society (IHS) and Barany Society (BS) [14, 16, 17], not reported by Holskin et al. [15]. Three different MABs were administered through subcutaneous injection according to manufacturer’s recommendations: Erenumab [14, 16, 17] Galcanezumab and Fremanezumab [16]. Hoskin et al. [15] did not report dose and administration modality (see Table 1). Endpoints evaluation was made clinically [14–16], with disease specific Patient Reporting Outcomes (PRO) measures [14, 16] and with instrumental tests (i.e. videonystagmography) [14]. Clinical evaluation comprised the report of migraine [14, 16] and dizziness [16] symptoms experienced in a month, and subjective evaluation of symptoms [15]. All authors reported significant improvement of VM symptoms after MAbs therapy [14–18], PRO measeures [14, 16, 17] and instrumental tests [14, 18]. The longer follow-up was conducted by Russo et al. with repeated evaluations at 3, 6, 9, 12 and 18 months [16], Inui et al. done a one year follow-up of the 42 years old female case report, Lovato et al. reported a 6 months follow-up (mean of 26.4 ± 2.1 weeks), Hoskin et al. did not report the follow-up time [15]. None of the authors reported adverse drug reactions. Detailed characteristics of included studies are depicted in Table 2.

**Table 2:** Characteristics and main findings of included studies

1st Author, year	Country	Study Design	Sample	Diagnosis	Evaluation	Drugs (dose)	Study period	Enpoints	JBI’s rating
Hoskin, 2022	USA	Retrospective cohort study	25	NR	Clinical	Erenumab Galcanezumab Fremanezumab Ubrogepant (NR)	NR	6 moderate improovment 9 significant improovment 6 mild improovment 4 no improovment	1/8
Russo, 2023	Italy	Prospective cohort study	50	BS-IHS	Clinical, MIDAS	Erenumab (140 mg) Fremanezumab (225 mg) Galcanezumab (120 mg)	78 weeks	mean MDD from 10.3 ± 1.9 to 0.7 ± 0.2. mean MIDAS scores from 52.8 ± 5.0 to 14.3 ± 3.2 mean MDM from 20.9 ± 1.6 to 9.3 ± 1 to 6.4 ± 1.2	4/8
Lovato, 2023	Italy	Retrospective cross-over cohort study	23	BS-IHS	Clinical, Videonistagmography, DHI	Erenumab (140 mg)	26.4 ± 2.1 weeks	mean MDM from 12.4 to 5.1 positional nystagmus from 47.8–4.3% of patients mean DHI from 30.2 ± 7.2 to 8.1 ± 3.1	5/8
Inui, 2023	Japan	Case report	1	BS-IHS	Videonistagmography, cVEMP, PTA, DHI, MIDAS, HIT	Erenumab (70 mg)	52 weeks	Improovment of Videonistagmography, cVEMP, PTA, DHI, MIDAS, HIT	NA
Sharon, 2020	USA	RCT protocol*	50	BS-IHS	VM-PATHI, DHI, MIDAS	Galcanezumab	NA	NA	NA

Abbreviation: BS-IHS (Barany Society and International Headache Society), cVEMP (cervical Vestibular Evoked Myogenic Potential); DHI (Dizziness Handicapi Inventory); HIT (Headache Impact Test); MDD (Monthly Days with Dizziness); MDM (Monthly Days with Migraine); MIDAS (Migraine Disability Assessment); NR (Not Reported); PROMIS SF (Patient-Reported Outcomes Measurement Information System Short Form); PTA (Pure Tone Audiometry); RCT (Randomized Control Trial), VM-PATHI (Vestibular Migraine-Patient Assessment Tool and Handicap Inventory).

*the study is ongoing, protocol available at https://clinicaltrials.gov/study/NCT04417361#contacts-and-locations.

### Quantitative Analysis

**Fig. 2:**
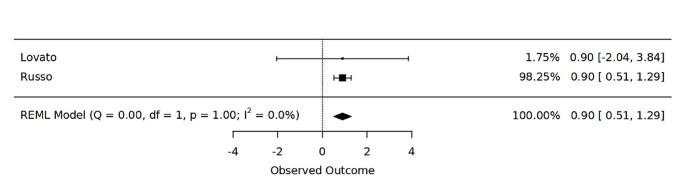
Forest plot of the effectiveness of CGRP-targeting monoclonal antibodies in reducing Migraine Disability Measurement (MDM) at 6 months following treatment

A meta analysis was possible for two studyes that reported mean days with migraine (MDM) changes after 6 months of therapy [14, 16]. The analysis revealed a statistically significant overall effect size of 0.9000 (Cohen’s d), with a 95% confidence interval ranging from 0.5114 to 1.2886. This signifies a substantial reduction in MDM scores, indicating decreased migraine-related disability among patients receiving CGRP-targeting monoclonal antibodies (see Fig. 2). The results were robust, with no significant heterogeneity observed between the included studies, as indicated by an I^2 value of 0.0000 and a *p*-value of 1.0000 for the Q statistic. Additionally, the Tau^2 value was negligible, suggesting minimal between-study variance.

## Discussion

The present rapid systematic review retrieved a case report [17] and three cohort studies with overall moderate [14, 16] to low [15] quality and a lack of published RCT related to MAbs therapy of VM. Moreover, a protocol for RCT was retrieved, with enrollment ending in July 2023 according to the last update made by the authors in February 2023 [18]. The published manuscript included in the qualitative analysis overall reported significant control of VM symptoms obtained by administering different types of anti-CGRP MAbs [14–17]. Nonetheless, a lack of adequate diagnostic criteria [15] and proper VM instrumental tools [15, 16] need to be stressed. Objective evaluation of VM symptoms including vestibular [14, 17] and audiometric tests [17], was performed only by two authors. Accordingly, the quantitative analysis was only feasible for migraine symptoms [14, 16] and revealed significant reduction in MDM at 6 months post-treatment with an overall pooled ES of 0.9. This suggests a substantial reduction in migraine-related disability among patients receiving CGRP-targeting MAbs, as expected [9]. Although the absence of severe adverse drug reactions is reported [14–16], a more comprehensive and nuanced safety profile, particularly concerning long-term use, remains unexplored. Additionally, the reporting of follow-up duration in some studies is incomplete [15], rendering the assessment of the duration of observed improvements challenging. The administration of different types of CGRP-targeting MAbs across the included studies (Erenumab; Ubrogepant; Galcanezumab and Fremanezumab) adds complexity and raises questions about the consistency of observed effects.

In the context of migraines, CGRP is recognized as a pivotal participant [1]. While the exact pathophysiology of VM remains incompletely understood, it shares similarities with migraines. Neuroanatomical connections between the vestibular system and brainstem nociceptive regions exists, with heightened signal transmission between these systems in VM patients [19, 20]. Migraine symptoms appear to be associated with changes in ion channel function, resulting in altered neural activity in the trigeminovascular system, leading to the release of neurotransmitters such as substance P and CGRP. Furthermore, CGRP receptors are expressed in the vestibular system and play a role in motion sickness. A recent study by Tian et al. induced a rat model of chronic migraine and vestibular dysfunction and observed elevated expression of CGRP, CLR, and RAMP1 in the vestibular nucleus [6]. Inhibiting the CGRP1 receptor attenuated mechanical allodynia, thermal hyperalgesia, and vestibular dysfunction. This was associated with changes in synaptic-associated proteins, restoration of synaptic ultrastructure, and a reduction in neuronal activation in the vestibular nucleus [6]. The PKC/ERK/CREB signaling pathway was implicated in CGRP-mediated regulation of synaptic transmission, and inhibiting the CGRP1 receptor reduced the expression of synaptic proteins and downstream phosphorylation of ERK and CREB, potentially contributing to central sensitization and synaptic transmission efficiency. The study also examined dendritic synapses and their ultrastructure, finding increased dendritic spine density, which was normalized by treatment. Moreover, levels of c-Fos, a marker of neuronal activation, were reduced by CGRP1 receptor inhibition. A recent preprint study by Rahman et al. suggested additional pathogenic roles for the CGRP pathway [21]. Behavioral tests revealed that CGRP administration, particularly in conjunction with vestibular challenges, induced anxiety-like behaviors and affected balance function in mice. This study suggested that targeting CGRP pathways could have therapeutic implications for these symptoms [21].

While monoclonal antibodies targeting the CGRP pathway have shown promise in the treatment of migraine disorders, they may be associated with side effects. Commonly reported adverse effects include injection site reactions, constipation, muscle spasms, and hypersensitivity reactions. Although these treatments are generally well-tolerated, understanding the full spectrum of potential side effects, especially with long-term use, remains crucial [22]. The advancements in CGRP-targeting therapies have introduced a new paradigm in the management of migraine, encompassing both injectable and oral formulations. Injectable CGRP monoclonal antibodies have been highlighted for their efficacy in reducing migraine days in adults, with specific dosages outlined to optimize patient outcomes (i.e. Erenumab 140 mg, Fremanezumab 225 mg, and Galcanezumab 120 mg administered once monthly). Moreover, the recent approval of Atogepant, an oral CGRP receptor antagonist, further broadened the treatment landscape, offering a non-invasive, well-tolerated option for the prophylaxis of episodic migraine, with dosages ranging from 10 mg to 60 mg daily [23]. A recent manuscript gave recommendations on the prescription of anti-CGRP monoclonal antibodies in children and adolescents [24]. The authors recommended usage for post-pubertal adolescents with frequent, moderate to severe migraine-related disability, after other preventive therapies have been unsuccessfully tried or contraindicated. Furthermore, the discussions emphasized the need for close monitoring and cautious application in this population, underscoring the ongoing requirement for empirical data to guide these treatments.

To the best of our knowledge, this is the first systematic review evaluating the use of MAbs targeting CGRP in the context of VM management. Several limitations merit consideration. Most notably, the paucity of available studies, particularly the absence of RCTs, impinges on the generalizability of the findings and underscores the necessity for further research. Furthermore, variability in the quality ratings of the included studies, with one study rated as low quality, introduces a potential source of bias and may influence the overall robustness of the evidence. The diversity in study designs, diagnostic criteria, and evaluation methods across the three included studies engenders heterogeneity. Lastly, the absence of a control group in the included studies limits the capacity to draw direct comparisons and attribute observed improvements solely to the MAb interventions.

## Conclusions

Our systematic review and meta-analysis indicate that monoclonal antibodies targeting CGRP hold promise for the treatment of Vestibular Migraine. While the available evidence is limited, it consistently demonstrates significant improvements in VM symptoms with the use of CGRP-targeting monoclonal antibodies. Our quantitative analysis specifically showed a noteworthy reduction in Monthly Days with Migraine after 6 months of treatment. This signifies a substantial decrease in migraine-related disability among VM patients treated with these antibodies. Importantly, no severe adverse reactions were reported in the reviewed studies. However, further research, including randomized controlled trials, is needed to comprehensively assess the efficacy on vestibular symptoms evalueted with proper instrumental tests and the long-term safety profile.
